# Study on Drying Control Strategy of White Radish Slice Based on Monitoring Medium Relative Humidity

**DOI:** 10.3390/foods11091197

**Published:** 2022-04-20

**Authors:** Dalong Jiang, Congcong Li, Zhian Zheng

**Affiliations:** 1 College of Engineering, China Agricultural University, Beijing 100083, China; bs20193070625@cau.edu.cn; 2 Department of Electronics, College of Information Science and Technology, Hebei Agricultural University, Baoding 071001, China; 3 Hebei Key Laboratory of Agricultural Big Data, Baoding 071001, China

**Keywords:** white radish, relative humidity control, color, hot air drying, energy

## Abstract

Enhancing the drying rate and dried products quality, as well as energy efficiency, is very tempting for the drying industry. Recently, a lot of investigations have illustrated that the drying temperature, air velocity, and sample thickness have significant influences on the drying process. However, few investigations took into account the relative humidity (RH) as an important hot-air-drying parameter in the drying process. Therefore, in the current work, white radish slabs were used to explore the drying characteristics and quality under the drying condition of a constant RH, decreasing the RH step by step and decreasing RH automatically, together with a constant air velocity of 1 m·s^−1^ and a drying temperature of 60 °C. Compared to continuous dehumidification, the step-down RH process was conducive to the material center temperature rise in the early stage of drying. When the material central temperature was increased and then the RH was reduced, the drying rate was increased and the overall drying time was shortened. The automatic-down RH control drying process includes three dehumidification processes. The respective RH control values were 40%, 30%, and 20% and the respective durations were 180 min, 90 min, and 60 min. The comprehensive quality evaluation showed that the comprehensive score of the automatic-down RH control process at 60 °C was the highest, which was 0.85. The L* and b* values of the automatic-down RH control were 26.0 and 1.67, respectively, which were better than those of the step-down RH, constant 20% RH, and constant 40% RH. The maximum rehydration ratio was 3.96 under the automatic-down RH control condition, and the quality was good. The lowest energy consumption under the condition of the automatic-down RH control was 0.90 kW·h·kg^−1^. The present work contributes to a better understanding of the effect of the RH on the drying characteristics and quality of white radish slices, which is useful for enhancing the drying rate and dried products’ quality as well as energy efficiency.

## 1. Introduction

In the past 20 years, the production of fruits and vegetables in China has developed rapidly, and the planting area and output are in the forefront of the world. However, most of fruits and vegetables have high water contents of 75%–95%, which are prone to corruption and deterioration, resulting in irreparable economic losses [1,2]. Among them, white radish (*Raphanus sativus* L.), also known as radish and cauliflower, belongs to cruciferous radish [3,4]. In 2017, the cultivation area exceeded 1.3 million hectares, with a total output of 45 million tons, becoming the second largest vegetable crop after Chinese cabbage in China [5]. Dried radish is popular among consumers due to its high edible value (rich polysaccharide content) and crisp taste. However, the moisture content of fresh radish is too high (up to more than 90%), which is particularly prone to browning, mildew and other problems. Natural drying time is long and is easily limited by climatic conditions.

Drying as one of the ancient food preservation methods can inhibit microbial reproduction and enzyme activity by reducing the moisture content of materials, thus reducing the deterioration of physical and chemical properties during storage, so as to prolong the shelf life and maintain the quality of products [6].

Among many drying technologies (vacuum drying, hot air drying, microwave drying, infrared drying, and radio frequency drying), hot air drying is widely used because of its simple structure and large batch processing capacity [7]. However, in the hot-air-drying technology, the hot air is both a heat carrier and a wet carrier. The heat required for the temperature rising and the water evaporation of the dried materials is provided by hot air, and the evaporated water is also taken away by hot air, which easily leads to a low energy utilization rate and a long drying time [8]. A large number of experiments showed that the drying temperature, wind speed, and chip thickness have significant effects on the drying rate (DR), whereas there was little research on the effect of the drying medium relative humidity (RH) on the DR and there was no unified conclusion [9,10,11,12].

Table 1 summarizes the research on the effects of RH on the hot air drying of different materials in recent years. In general, higher RH can accelerate the increase of the material center temperature, thus accelerating the movement of water molecules in the material [13]. Using high-RH air to preheat the fresh sample can make the temperature and moisture content of the center and the surface close to each other and then reduced the humidity of the medium, so that the temperature gradient from the center to the surface of the fresh sample is consistent with the humidity gradient, which is conducive to the acceleration of the drying and the reduction of the crusting phenomenon. Nevertheless, there was no real-time measurement and analysis of the change law of the RH and material temperature of the drying medium. The keeping time of high RH and the time of moisture removal are determined by experience or a lot of experiments, and the influence of the medium RH change on the material state was not revealed. Moreover, the change of the medium RH in the drying process is closely related to the material state. Therefore, it is necessary to propose an adaptive drying strategy based on monitoring the RH of the medium.

To sum up, this paper put forward an automatic adjustment control strategy of the medium RH in the drying process and analyzed the effects of different constant RH, step-down RH, and automatic dehumidification conditions on the drying characteristics, product color, rehydration rate, energy consumption, and polysaccharide content of white radish. This study provides a theoretical basis for realizing the intelligent industrial drying of white radish.

## 2. Materials and Methods

### 2.1. Materials

White radish (*Raphanus sativus* L.) was used as a raw material and purchased from local farmers’ market in Beijing. Fruits with a uniform size (the average diameter was 6 ± 1 cm, and the length was 23 ± 1 cm) and without visual damage were selected, washed and cut in slices. The initial moisture content on a wet basis was 94.5% ± 0.8% (drying method [24,25]; temperature: 105 °C; period: 24 h). Triplicate measurements were conducted. The white radish was cut into round slices with a diameter of 6 ± 0.2 mm, and the batch weight was about 5 kg. Before the experiment, they were stored in a refrigerator at 4 ± 1 °C.

### 2.2. Main Experiment Equipment and Procedure

**Figure 1:**
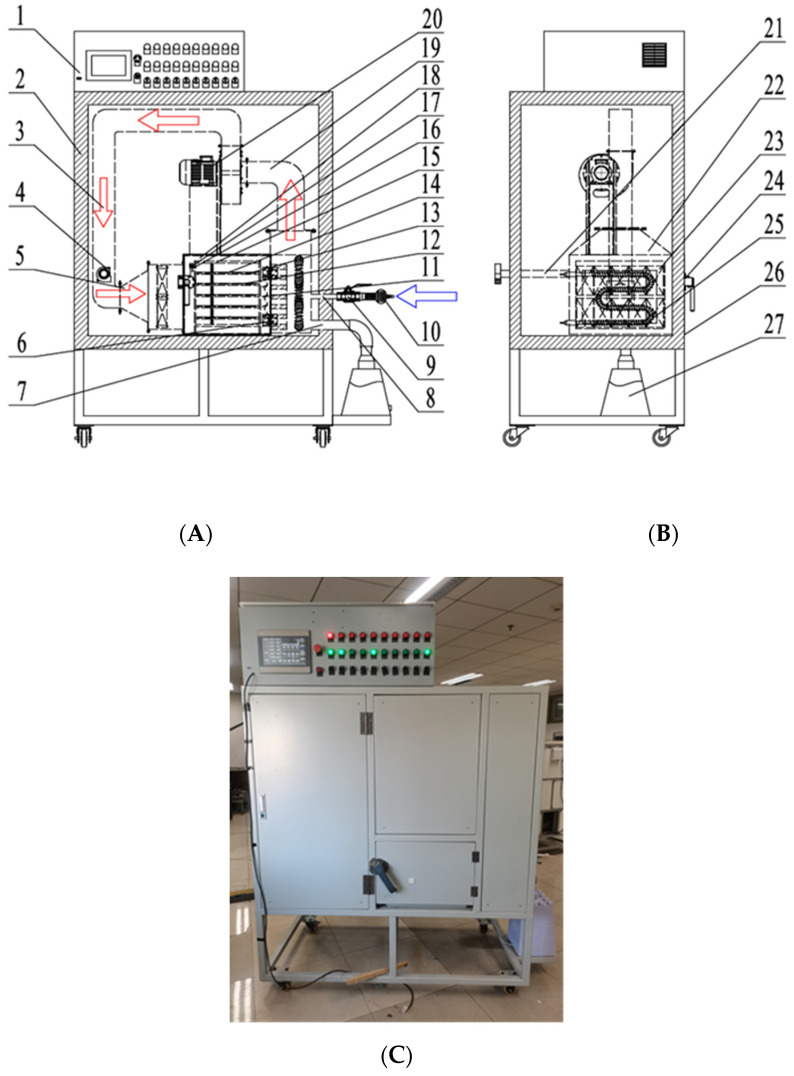
Schematic diagram of the overall structure of a multi-layer box-type hot air dryer based on temperature and humidity control: (**A**) main view of dryer; (**B**) left view of the dryer; (**C**) physical drawing of the dryer. 1. control system; 2. insulation layer; 3. air inlet duct; 4. dehumidification fan; 5. bell mouth; 6. door hinge; 7. humidification port; 8. air inlet port; 9. manual ball valve; 10. dehumidification devices; 11. thrusting needle PT100 temperature sensor; 12. tray; 13. carbon-crystal infrared heating plate; 14. surface mount PT100 temperature sensor; 15. material rack; 16. SHT35 temperature and humidity sensor; 17. probe-type PT100 temperature sensor; 18. drying chamber; 19. return air duct; 20. centrifugal blower; 21. drainage pipe; 22. heating chamber; 23. axial flow fan; 24. door handle; 25. W-shaped finned heating tube; 26. drying chamber door; 27. ultrasonic humidifier.

A multi-layer box-type hot air dryer installed in the College of Engineering of China Agricultural University, Beijing, China was used in the present study. A schematic diagram of the hot-air-drying equipment is shown in Figure 1. The drying equipment mainly consisted of a hot air circulation motor, a drying chamber, a gas-collecting chamber, a material rack, an electric heating pipe, a dehumidification fan, an insulation layer, and an automatic control system. In the vertical direction, there were three drying layers of the same size. A proportional–integral–derivative (PID) controller (Omron, model E5CN, Tokyo, Japan) with an accuracy of ±0.1 °C was used to control the air temperature in the drying chamber. The hot air circulation motor drove the wind blades to rotate to drive the airflow. The airflow entered the heating chamber after contacting with the materials through the gas-collecting chamber and then was reheated by the electric heating pipe, and finally, the hot air circulation was realized through a return air channel. A dehumidification fan was connected with a trumpet-shaped air inlet through a dehumidification duct to realize the moisture removal in the drying process.

**Figure 2:**
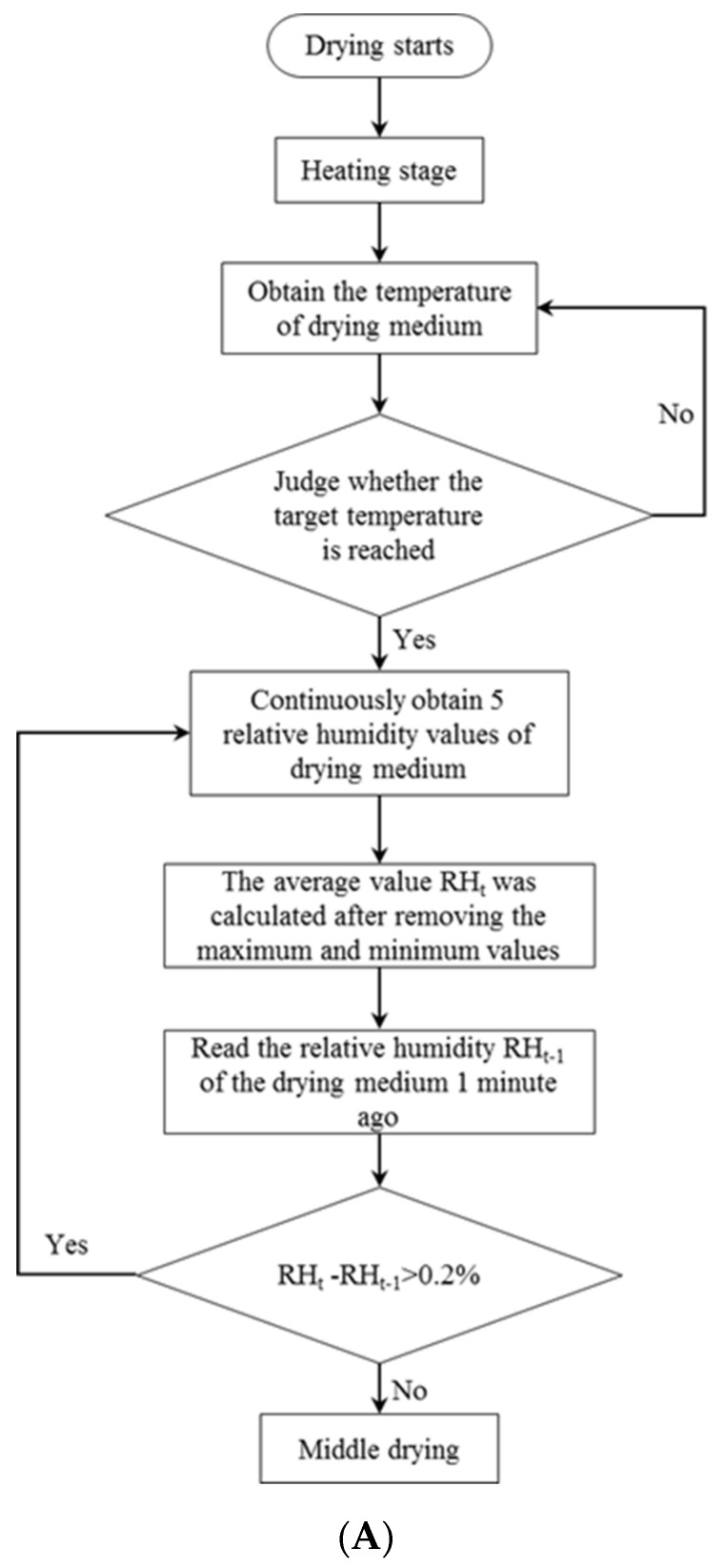

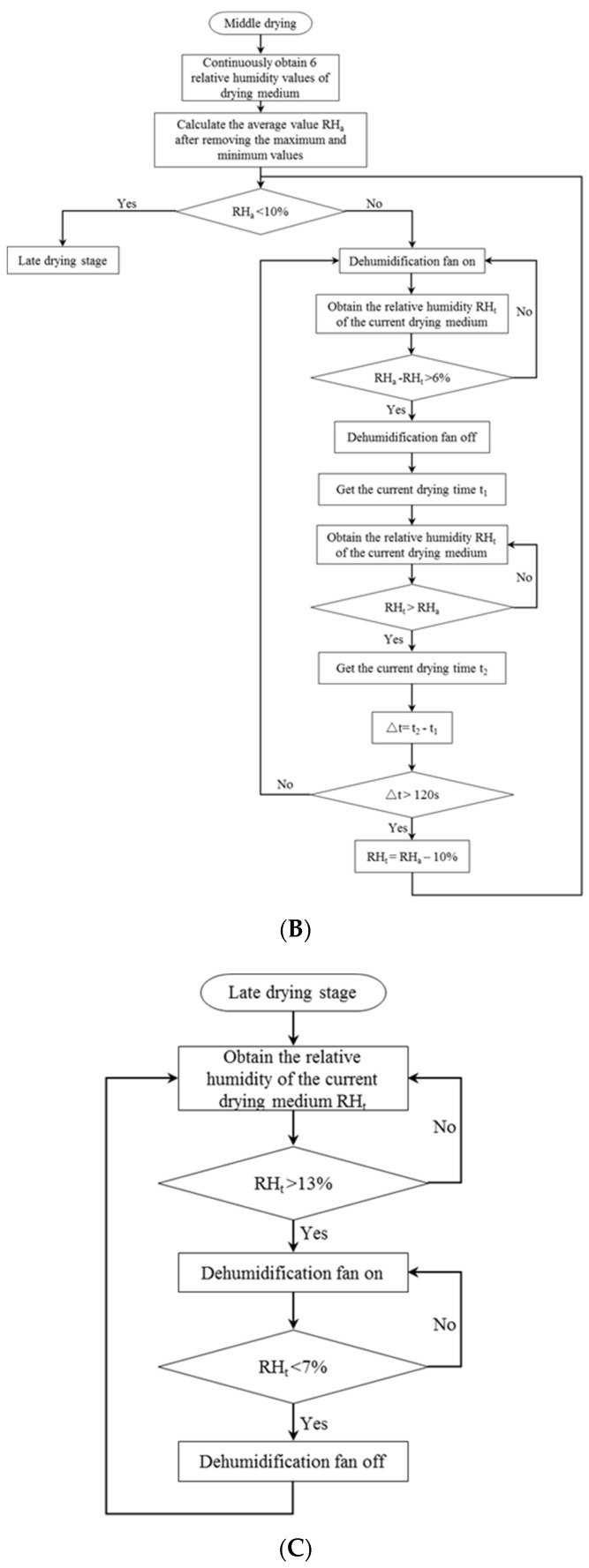
Flow chart of the control procedure: (**A**) control flow chart in the early drying stage; (**B**) control flow chart in the middle drying stage; (**C**) control flow chart in the late drying stage.

The automatic dehumidification control program was divided into three stages according to the RH change of the drying medium at the air inlet of the drying chamber obtained from the pre-experiments: early drying, middle drying, and late drying. The specific control flow was shown in Figure 2.

Other experiment instruments and equipment include the following devices: 

an EU-2600A UV-visible spectrophotometer (Shanghai Rangqi Instrument Technology Co., Ltd., Shanghai, China); a DHG-9140A electric constant temperature blast drying oven (Shanghai Yiheng Technology Co., Ltd., Shanghai, China); an SMY-2000SF colorimeter (Beijing Shengmingyang Technology Development Co., Ltd., Beijing, China); an electronic constant-temperature stainless steel water bath pot (Shanghai Yichang instrument yarn screen factory, Shanghai, China); a machine vision system (industrial camera BASLER ACA2500-14GC; a CMOS photosensitive chip); an electronic balance (Ohaus International Trade Shanghai Co., Ltd., Shanghai, China, 0.001 g); an electronic balance (Shanghai Co., Ltd., Shanghai, China, 0.001 g); DTS7178 three-phase four-wire electronic watt-hour meter (Shanghai Minrong Electric Co., Ltd., Shanghai, China); several dryers.

### 2.3. Experiment Method

The multi-layer box-type hot air dryer was turned on to preheat to the set temperature and RH. The sliced radish slices were evenly spread in seven trays (500 × 300 mm), and the loading capacity of each tray was about 800 ± 50 g. Three white radish slices were randomly taken from a material tray, and the temperature sensor PT100 (Beijing Youpusi science and Technology Center, Beijing, China) was inserted into the center of the white radish slices. The rest of the trays were used to measure the weight change regularly. During the process, the energy consumption in the drying process was monitored by the watt-hour meter in real time. Considering the loss of hot air in the weighing process, which interfered with the monitoring of the temperature of the material center and the humidity of the drying medium, a heat insulation board was added at the entrance of the drying chamber, and the process from taking it out to putting it in of the tray only took 20 s. Previous studies [14,15] have shown that the drying quality of white radish was better when the drying temperature was 60 °C, so the parameters of this experiment were set as shown in Table 2. The fan controller frequency was set at 30 Hz, the speed was 1700 rpm, the wind speed was 1 m·s^−1^, and the medium RH control threshold was set to 3%. At the end of drying, the moisture content on the wet basis was 8%, that is, the moisture content [24,25] on the dry basis was 0.087 g·g^−1^.

### 2.4. Calculation Method of Experiment Parameters

#### 2.4.1. Calculation Method of the Moisture Ratio (MR) and the DR

The drying characteristic curve was the curve of the material moisture content changing with time in the process of hot air drying at a certain temperature, RH, and flow rate. The calculation equation of the dry basis moisture content (*M_t_*) was as follows [18,26,27]:

(1) $$M_{t}=\frac{W_{t}-G_{d}}{G_{t}},$$

where *W_t_* indicates the total mass of the material at the current time (g); *G_d_* indicates the dry matter mass of the material (g); *M_t_* indicates the dry basis moisture content of the material at the current time (g·g^−1^).

The calculation equation of the MR was as follows [18,26,27]:

(2) $$MR=\frac{M_{t}-M_{e}}{M_{0}-M_{e}},$$

where *M_e_* indicates the equilibrium dry basis moisture content of the material, (g·g^−1^); *M*_0_ indicates the initial dry basis moisture content of the material (g·g^−1^). Since the moisture content of the balanced dry base was far less than that of the dry base of the material in the drying process, its value can be ignored. The equation for calculating the MR can be simplified as follows:

(3) $$MR=\frac{M_{t}}{M_{0}}.$$

The equation of the DR was as follows [18,26,27]:

(4) $$DR=\frac{M_{t1}-M_{t2}}{t_{2}-t_{1}},$$

where *t*_1_ and *t*_2_ indicate the drying time (h); *DR* indicates the drying rate between drying times *t*_1_ and *t*_2_ (g·(g·h)^−1^).

#### 2.4.2. Determination of the Color, RR, and Energy Consumption

The CIE L^*^a^*^b^*^ color system can accurately expressed the actual color perceived by human eyes and can distinguish lightness and color information quantitatively. An SMY-2000SF color difference meter (Beijing Shengmingyang Science and Technology Development Co., Ltd., Beijing, China) was used to determine the L* (brightness) and b* (yellow and blue value) of white radish tablets after drying. The determination method was described in the literature [28,29,30]. The dominant color of white radish slices was white. The larger L* was and the smaller b* was, the whiter the white radish slices and the better the quality of white radish slices [28,29,30]. The total color differences between fresh and dried white radish slices (ΔE) were calculated according to the following equation [31]:

(5) $$\Delta E=\sqrt{{\left(a{*}_{0}-a{*}_{1}\right)}^{2}+{\left(b{*}_{0}-b{*}_{1}\right)}^{2}+{\left(L{*}_{0}-L{*}_{1}\right)}^{2}},$$

where a*_0_ is the degree of redness/greenness of fresh white radish slices, b*_0_ is the degree of yellowness/blueness of fresh white radish slices, L*_0_ is the degree of lightness/darkness of fresh white radish slices, and a*_1_, b*_1_, and L*_1_ are the color values of dried white radish slices.

Dry products generally need to be rehydrated, before they can be eaten. The degree to which dry products recover to the original fresh state after rehydration is an important index to measure the quality of dry products, which is usually measured by the rehydration ratio (RR). The dried radish (8.15 ± 0.61 g) for each RH control method was immersed into distilled water at 40 °C for 60 min and then placed on an absorbent paper for 20 min [28,29,30]. The drained samples after rehydration were weighed, and the RR was calculated by Equation (6):

(6) $$RR=\frac{G_{f}}{G_{g}},$$

where *G_f_* indicates the weight of radish after rehydration by draining the water (g); *G_g_* indicates the dried radish weight (g).

The measurement of energy consumption was calculated by the following equation [32,33]:

(7) $$Q=\frac{1000W}{m_{i}\times \left(\varphi_{0}-\varphi_{i}\right)},$$

where *Q* indicates the unit energy consumption of this experiment (kW·h·kg^−1^); *W* indicates the difference between the watt-hour meter readings before and after this experiment (kW·h); *m_i_* indicates the difference between the watt-hour meter readings before and after the experiment (g); *φ_i_* indicates the moisture content of the wet base at the end of the material (%); *φ*_0_ indicates the initial wet base moisture content of the material (%).

#### 2.4.3. Determination of the Total Sugar Content

As described by Macedo et al. [34], the phenol-sulfuric acid method was used to determine the total sugar content in dried white radish slices. The results of each group were repeated 6 times, and the freeze-dried group was used as the control for significance analysis.

#### 2.4.4. Comprehensive Evaluation Method

In order to unify the data, it was necessary to normalize the evaluation index. The normalization equations of positive indicators (RR, total sugar content, and L*) and negative indicators (drying time *t*, energy consumption *Q*, and b*) were as follows [35]:

(8) $$y_{i}=\frac{x_{i}-x_{\min}}{x_{\max}-x_{\min}},$$

(9) $$y_{i}=\frac{x_{\max}-x_{i}}{x_{\max}-x_{\min}},$$

where *y_i_* indicates the normalized value; *x_i_* indicates the actual value of the index; *x*_max_ and *x*_min_ indicate the maximum and minimum values of the index, respectively. The comprehensive score of drying conditions was obtained by weighting according to Equation (10) [35]:

(10) $$Y=y_{1}l_{1}+y_{2}l_{2}+y_{3}l_{3}+y_{4}l_{4}+y_{5}l_{5}+y_{6}l_{6},$$

where *y*_1_, *y*_2_, *y*_3_, *y*_4_, *y*_5_, and *y*_6_ indicate the normalized results of the RR, brightness, drying time, energy consumption, and yellow-blue value, respectively, and *l*_1_, *l*_2_, *l*_3_, *l*_4_, *l*_5_, and *l*_6_ are their respective weights. To guarantee the condition of a good RR, total sugar content, and L*, the energy consumption (*Q*) and drying time (*t*) were reduced at the same time. Using the analytic level method [35], the weights of the RR, total sugar content, L*, drying time (*t*), energy consumption (*Q*), and yellow–blue value b* were 0.2, 0.3, 0.2, 0.1, 0.1, and 0.1, respectively.

### 2.5. Statistical Analyses

The data were analyzed using the statistical software Matlab2017a. Significance was defined at *p* ≤ 0.05 by the least significant difference (LSD) multiple comparison test. Each experiment was repeated three times.

## 3. Results and Analysis

### 3.1. Effect of the Constant RH and the Step-Down RH on Hot-Air-Drying Characteristics of White Radish Slices

**Figure 3:**
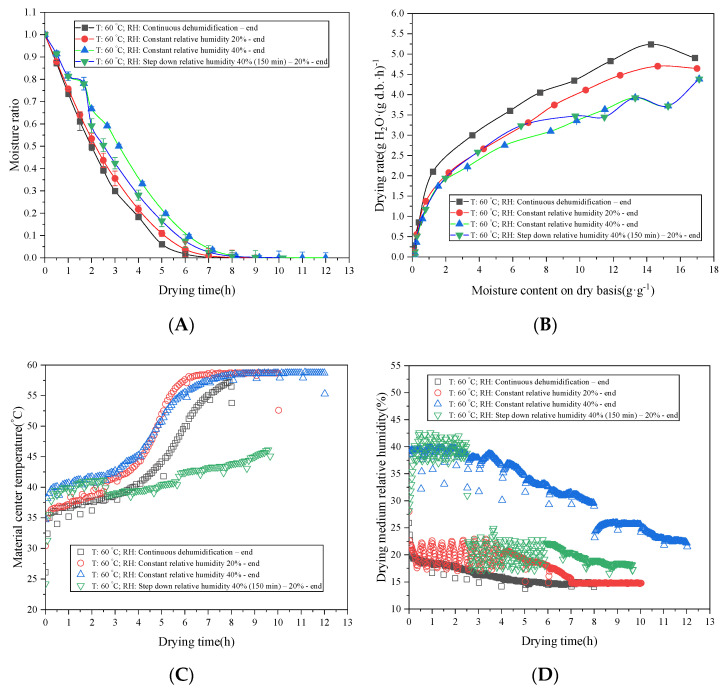
Drying characteristic (**A**), drying rate (**B**), material central temperature (**C**), and drying medium RH value (**D**) of white radish as a function of time under constant RH and step-down RH conditions. Note: velocity at 1.0 m·s^−1^.

The hot-air drying characteristics and DR curves of radish slices under different constant RH values and step-down RH values are shown in Figure 3. At a constant drying temperature of 60 °C, the drying times of continuous dehumidification, the constant RH of 20%, the constant RH of 40%, and the step-down RH were 8.9, 12.0, and 10.17 h, respectively. Under the constant RH control, the drying time decreased with the decrease of RH. According to Fick’s law and its boundary conditions, the lower the environmental RH was, the greater the water vapor pressure difference between the material and the environment was and the higher the DR was. Therefore, reducing RH was conducive to shortening the drying time and improving the drying efficiency [21].

For the step-down RH process, that is, the DR was significantly increased when RH was kept at 40% (150 min) in the initial drying period and then decreased to 20% (Figure 3B), and the drying time was between 20% RH and 40% RH. Figure 3C shows that the higher the RH was in the early drying stage, the faster the material central temperature rose. According to he moisture enthalpy diagram of air, when the dry bulb temperature was fixed, the higher the RH was, the higher the wet bulb temperature was and the higher the enthalpy value was [36]. According to Fourier’s law of thermal conductivity, the higher the enthalpy of the drying medium was, the faster the material heated up. Therefore, the material center temperature of early material rose fastest and reached the highest temperature at 40% RH (about 45.2 °C). After the end of preheating, part of the heat was used to evaporate water, part was used to heat the material, and the final material temperature and environmental temperature tended to balance. In the early stage of the step-down RH drying process, the material center temperature was relatively high. After 150 min, the temperature dropped rapidly from 43 °C to 37.5 °C. This was due to the decrease of ambient RH, the increase of water vapor pressure, and the rapid evaporation of moisture on the surface of the material heat absorption, leading to a sudden drop in the material central temperature. This was also consistent with the law that the DR increased rapidly at the RH jump point (Figure 3B). The variation trend of the temperature curves under different RH was consistent with the research results of Li et al. [37] and Barati et al. [13].

Under the different constant RH and step-down RH control conditions, the variation rules of RH in the dry medium were different. The RH of the drying medium decreased rapidly with the drying process under the continuous dehumidification condition (Figure 3D). The RH of the drying medium showed a trend of a rapid increase and a gradual decrease, reaching its maximum value at the node of 30 min (about 43%) under the constant RH of 40%. The RH of the drying medium was controlled by the evaporation rate of the material at a constant drying temperature of 60 °C. As the DR decreased gradually, the RH in the drying chamber could not be maintained at 40% and decreased slowly until it was close to the moisture content of the ambient air. A similar trend was observed at the constant RH of 20%.

In the step-down RH control process, the RH of the drying medium fluctuated within the threshold range of the control value in the early stage of drying. When the RH control value dropped from 40% to 20%, the RH of the drying medium directly dropped from 43% to 16%, which also confirmed the rapid increase of the DR at this time node (Figure 3B). The RH of the drying medium began to decrease slowly, until it was close to the moisture content of ambient air for a drying time of 6 h. Compared with that at the constant RH of 20%, the drying time of the last active dehumidification point was extended by 1 h. The RH of the drying medium had a significant influence on the material temperature rise and the DR. By analyzing the effect of the step-down humidity control on the material-drying characteristics, it was shown that a reasonable monitoring drying medium RH was of great significance to shorten the drying time.

### 3.2. Effect of the Automatic-Down RH on the Hot-Air-Drying Characteristics of White Radish Slices

**Figure 4:**
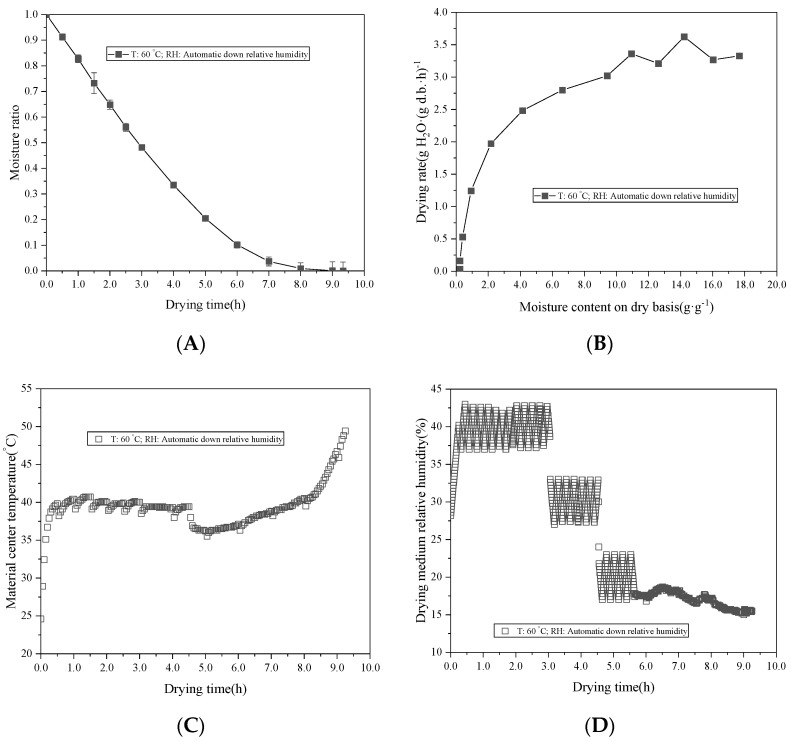
Drying characteristic (**A**), drying rate (**B**), material central temperature (**C**), and drying medium RH value (**D**) of white radish as a function of the drying time under automatic-down RH conditions. Note: velocity at 1.0 m·s^−1^.

The MR showed an exponential decline with the extension of the drying time (Figure 4A). The DR in the early drying stage was small, and there were two obvious fluctuations. This was due to the high RH of the medium in the early stage of drying, the small difference of water vapor partial pressure between the material surface and the environment, and the slow water evaporation. When the medium RH changed, the moisture evaporation increased rapidly, resulting in a significant increase in the DR (Figure 4B). Due to the high RH of the medium and the high enthalpy value [21,33], the material center temperature rose rapidly, and the temperature reached the high value (about 41.8 °C) at the time node of 1 h. Part of the heat transferred by the medium was used for the material temperature rising, the other part was used for moisture evaporation, and a small part of heat was lost. This also explained that the material temperature always fluctuated around 40 °C before the time node was 5 h, the moisture evaporation of the material decreased in the later stage of drying, and the temperature of the material center rose rapidly (Figure 4C). As shown in Figure 4D, the drying process consisted of three dehumidification processes lasting 180 min, 90 min, and 60 min, respectively. In the early stage of drying, the medium RH rapidly increased to the high value (about 42.9%) at the time node of 20 min. The system controlled the medium RH value at about 40%. The high RH environment slowed down the moisture removal rate but promoted the rapid rise of the material center temperature. The system judged that the medium humidity could not be maintained at 40% and dropped to 30% at the time node of 3 h. Similarly, the system determined that the medium RH could not be maintained at 30% and dropped to 20% at the time node of 4.5 h. The system was adjusted for continuous moisture removal, and the final RH was maintained at about 15% at the time node of 5.8 h. The drying time was greatly shortened, and the drying efficiency was improved by using the automatic RH control drying method.

### 3.3. Comparison of the Color, RR, Total Sugar Content, and Energy Consumption

**Figure 5:**
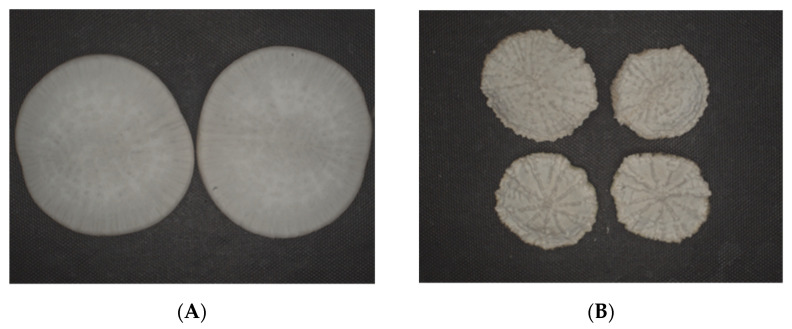

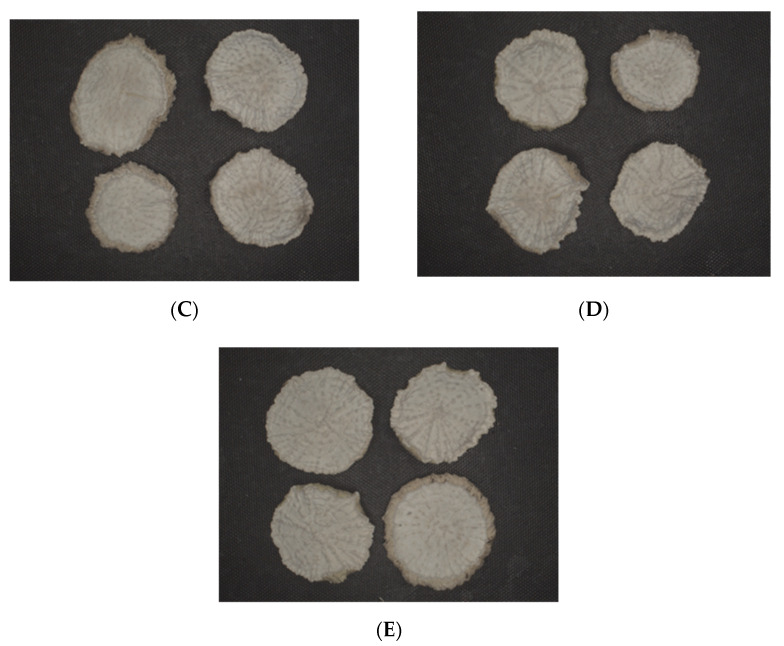
Overall appearances of white radish slices under different drying conditions: (**A**) control; (**B**) temperature of 60 °C and constant RH of 20% to the end; (**C**) temperature of 60 °C and constant RH of 40% to the end; (**D**) temperature of 60 °C and step-down RH values of 40% (150 min) and then 20% to the end; (**E**) temperature of 60 °C and automatic-down RH.

The determination results of the total sugar content, RR, brightness L*, drying time t, energy consumption Q, and yellow–blue value b* under different drying conditions are shown in Table 3, and the physical comparison is shown in Figure 5. The results showed that L* and b* values of the automatic RH control process conditions were 26.0 and 1.67, respectively, which were better than the conditions of the step-down RH, constant 20% RH, and constant 40% RH. There were also significant differences in the color difference values of dry products under different drying conditions, and the color difference value of the automatic humidity control group was the smallest, which was 3.61. It may be that the drying time was short, which was conducive to the preservation of the color of white radish slices. Under the constant 40% RH condition, browning was easy to occur due to the activity of water and enzyme and oxidation.

The RR of dry products to a certain extent reflected the structure of materials in the drying process and the degree of cell damage [38]. There was no significant difference in the RRs under different conditions of the constant RH, step-down RH, and automatic RH control, but the maximum RR was 3.96 under the condition of the automatic RH control. It showed that the drying intensity of the automatic RH control program was mild and the material structure deformation was small.

In order to compare the energy consumption of each process condition more clearly and intuitively, it was assumed that the energy consumption at the constant RH of 40% was 1 and the energy consumption under other drying conditions was the ratio. The energy consumption of the step-down RH controlled process was between the constant 20% RH and the constant 40% RH, which was 0.95. The lowest energy consumption under the condition of the automatic RH control was 0.90, which was 7.21%, 10.00%, and 5.26% lower than those of the constant 20% RH, constant 40% RH, and step-down RH control process conditions, respectively. Therefore, the reasonable control of the medium RH in the drying process was conducive to shortening the drying time and reducing energy consumption.

The total sugar content is one of the important nutrients in dried radish products, which directly affects the taste and flavor of consumers. Different drying conditions had significant (*p* < 0.05) effects on the total sugar content of dried white radish slices, and the total sugar content of dried products in the fresh group was the highest (45.01%). The total sugar content of dried products under the drying condition of the constant humidity of 40% was significantly lower than under other drying conditions, which was 25.54%. The total sugar content of white radish slices dried by continuous dehumidification and automatic RH control was high. It indicated that the short drying time was conducive to the retention of more total sugar content in dried products and the long drying time led to the accumulation of heat in the material, which promoted the degradation of polysaccharides [39,40].

### 3.4. Comprehensive Evaluation of Different Drying Methods

Table 3 indicates that the highest comprehensive score was 0.85 under the condition of the automatic RH control process at 60 °C. In the early stage of drying, maintaining a high RH in the “wetting section” made the material rapidly heat up. When the material temperature reached the wet-bulb temperature and became stable, the “wetting section” increased the water vapor pressure difference between the material and the environment and increased the DR. The “low wetting section” further promoted the removal of moisture inside the material. It could reduce the energy consumption, shorten the drying time and obtain better product quality by adjusting the RH in the drying process reasonably. For example, the Δ*E* of dry products in the automatic humidity control group was significantly (*p* < 0.05) lower than those of the other groups, while the Δ*E* at the 40% high humidity maintained for a period of time was significantly (*p* < 0.05) higher than those of the other groups. In addition, the automatic humidity control group had the highest score, which was significantly (*p* < 0.05) higher than the other experimental groups. This study provides theoretical guidance for the realization of high-efficiency, energy-saving and quality-drying of intelligent industrial-grade hot-air-drying equipment.

## 4. Conclusions

In this study, white radish slices were dried by hot air with different medium RH control strategies. The main conclusions were as follows:

(1) The step-down RH process was conducive to the material temperature rise in the early stage of drying process. When the material central temperature was increased and then the RH was reduced, the DR was increased and the drying time was shortened.
(2) The drying time was greatly shortened, and the drying efficiency was improved by using the automatic RH control drying method. The drying process includes three dehumidification processes. The respective RH control values were 40%, 30%, and 20%, and the respective durations were 180 min, 90 min, and 60 min. The drying time using automatic RH control was 1 h shorter than that of the step-down RH.
(3) Comprehensive evaluation of RR, brightness L*, drying time *t*, energy consumption *Q*, and yellow–blue value b* showed that the L* and b* values of the automatic RH control process conditions were 26.0 and 1.67, respectively, which were better than the conditions of the step-down RH, constant 20% RH, and constant 40% RH. There was no significant difference in the RRs under different conditions of the constant RH, step-down RH, and automatic RH control, but the maximum value of the *RR* was 3.96 under the automatic RH control condition. The lowest energy consumption under the condition of automatic RH control was 0.90.

## Figures and Tables

**Table 1:** Research status of the relative humidity (RH) in hot air drying.

Materials	Main Conclusion	Author
Pods	Appropriate relative humidity could improve the color, shape, and rehydration rate of dried pods.	Jia et al. [14]
Rice and fish noodles	The high-temperature and high-RH drying technology could improve the drying rate and the quality.	Liu et al. [15]
Rice	Suitable drying conditions of high temperature and high RH could improve the moisture effective diffusion coefficient and reduce the drying energy consumption.	Zhao et al. [16]
Cherry tomatoes	Staged variable temperature and humidity drying process could effectively reduce the loss of nutrients and color changes of cherry tomatoes and shorten the drying time.	Wang et al. [17]
*Panax notoginseng* roots	The step-down relative humidity strategy contributed to the formation of a porous structure, enhancement of drying efficiency, and quality improvement.	Jiang et al. [18]
Shiitake mushrooms	The high RH had a negative effect on the flavor components of shiitake mushrooms.	Li et al. [19]
Hawthorn	The color difference, Vitamin C content, and sensory scores of dried products were better under the conditions of a constant RH of 30% and phase humidification (RH of 50% at the constant rate period and RH of 30% at the falling rate period).	Liu et al. [20]
Carrot slabs	Taking the rehydration ratio, color value, drying time, and energy consumption into account, conditions of 50% RH kept for 30 min and then RH reduced to 20% were proposed as the favorable condition for drying carrot slabs.	Ju et al. [21]
Carrot	The step-down RH could accelerate the drying rate to prevent surface casehardening in the porous agriculture products of which surfaces were easily crusted during drying.	Ju et al. [22]
Carrot	The quality of dried carrot was related to the high humidity maintenance interval and the drying time. In order to improve the quality of dried carrot, a longer humidification time and a lower humidity of moisture should be selected.	Zhou et al. [23]

**Table 2:** Experimental design and experimental parameters.

Process	Experimental Number	Drying Temperature (°C)	RH (%)	Stage Time (h)
Constant RH	1	60	20	To the end
	2		40	To the end
Step-down RH	3	60	40	150 min
			20	To the end
Automatic-down RH	4	60	-	To the end

**Table 3:** Color values, rehydration ratios, energy consumptions, drying times, total sugar contents, and comprehensive scores under different drying conditions.

*No*	L* White Black	b* Yellow–Blue Value	Δ*E*	Rehydration Ratio (g·g^−1^)	Energy	Drying Time (h)	Total Sugar Content (%)	Comprehensive Score
Fresh	21.1 ± 0.03 ^c^	–0.19 ± 0.05 ^e^	-	-	-	-	45.01 ± 0.03 ^a^	-
1	16.19 ± 0.07 ^e^	3.08 ± 0.06 ^b^	7.32 ± 0.03 ^b^	3.86 ± 0.04 ^a^	0.97	9 ± 0.05 ^d^	37.29 ± 0.01 ^b^	0.58 ^c^
2	19.28 ± 0.02 ^d^	3.75 ± 0.04 ^a^	15.24 ± 0.02 ^a^	3.89 ± 0.02 ^a^	1	12 ± 0.02 ^a^	25.54 ± 0.02 ^e^	0.49 ^d^
3	25.1 ± 0.01 ^b^	2.83 ± 0.03 ^c^	4.17 ± 0.01 ^c^	3.88 ± 0.03 ^a^	0.95	10.17 ± 0.10 ^b^	31.17 ± 0.03 ^d^	0.73 ^b^
4	26.0 ± 0.02 ^a^	1.67 ± 0.01 ^d^	3.61 ± 0.07 ^d^	3.96 ± 0.01 ^b^	0.90	9.17 ± 0.24 ^c^	35.89 ± 0.01 ^c^	0.85 ^a^

Note: Different letters ^a–e^ indicate the significant differences (*p* < 0.05) of different drying methods; the energy consumption under the constant 40% RH drying condition was 7.13 kW·h·kg^−1^.

## Data Availability

All the results showed in the manuscript could be requested to the corresponding author who would provide them.
